# Assessment of sagittal spinopelvic alignment in asymptomatic Chinese juveniles and adolescents: a large cohort study and comparative meta-analysis

**DOI:** 10.1186/s13018-021-02773-z

**Published:** 2021-11-02

**Authors:** Canglong Hou, Kai Chen, Yu Chen, Tianjunke Zhou, Mingyuan Yang, Ming Li

**Affiliations:** 1 Department of Orthopedics, Shanghai Changhai Hospital, Shanghai, 200433 China; 2 Department of Orthopedics, Huashan Hospital, Fudan University, 200040 Shanghai, China; 3 Basic Medicine College, Navy Medical University, Shanghai, 200433 China

**Keywords:** Chinese, Juvenile, Adolescent, Sagittal alignments, Meta-analysis

## Abstract

**Study design:**

Retrospective study and comparative meta-analysis.

**Objective:**

To document the sagittal spinopelvic alignment in a large cohort study in asymptomatic Chinese juveniles and adolescents, and to explore whether these parameters were different from various regions using meta-analysis.

**Methods:**

Medical records of 656 asymptomatic Chinese juveniles and adolescents were reviewed, whose mean age was 13.14 ± 3.41 years old, including 254 male and 402 female volunteers. Demographic and lateral radiological parameters were evaluated. Furthermore, a systematic online search was performed to identify eligible studies. Weight mean difference (WMD) with 95% confidence interval (CI) were used to evaluate whether these sagittal parameters were different from various regions.

**Results:**

The mean value of sagittal spinopelvic alignment in this study was calculated and analyzed respectively. Significant differences of PI (34.20 ± 4.00 vs. 43.18 ± 7.12, *P* < 0.001) and PT (3.99 ± 6.04 vs. 8.42 ± 7.08, *P* < 0.001) were found between juveniles and adolescents. A total of 17 studies were recruited for meta-analysis. For juvenile populations, TK, PI and SS of Caucasians were significantly larger than those of our study (all *P* < 0.001). As for adolescent populations, PI (*P* = 0.017), TK (*P* = 0.017) and SS (*P* < 0.001) of Caucasians was found to be greater when compared with that of our study. All in all, TK, PI and SS in Chinese pre-adult populations were significantly smaller than those populations in Caucasian regions (all *P* < 0.001).

**Conclusion:**

Our study was the first large-scale study that reported the mean values of sagittal parameters in asymptomatic Chinese juveniles and adolescents. There were significant differences in TK, PI and SS between our study and other previous reported populations, which reminded us for using specific mean values in different populations when restoring a relatively normal sagittal spinopelvic balance in spinal deformity.

## Introduction

Sagittal alignment of the spine and pelvis is getting an increasing recognition of importance since the association between sagittal alignment and HRQoL (Health of Related Quality of Life) has been verified in many studies [1–3]. Therefore, how to restore the sagittal alignment is an important aspect to consider in the evaluation and treatment of spinal pathologies [4].

Assessments of normal mean values of sagittal parameters in juveniles and adolescents are the key to the restoration of sagittal alignment in correction surgery. However, although the normal mean values of sagittal parameters in juveniles and adolescents have been reported in many studies, the results were conflicting [4–20]. In addition, it has been verified that sagittal spinopelvic parameters vary in various ethnicities. For example, the normal mean value of TK (Thoracic kyphosis), LL (lumbar lordosis), PI (pelvic incidence), PT (Pelvic tilt) and SS (sacrum slope) in Chinese adolescents reported by Qiu et al. [5] were 20.8°, 49.3°, 44.6°, 11.3° and 33.3°, respectively, which was consistent with Wang et al.’s [21] and Zhu et al.’s study [4]. However, these sagittal parameters in Caucasians [14] were significantly greater than those in Chinese populations, with TK of 28°, LL of 55°, PT of 8° and SS of 37°. Measured postural angles are variables that can be measured to quantify posture. Like spinopelvic parameters, measured postural angles also vary differently in various countries and ethnicitie [22]. In addition to the various parameters in different populations, sagittal spinopelvic parameters also play important roles in growth and development of spine and pelvis. Diebo et al.’s study [23] suggested that compensation for sagittal was ethnicity dependent and these different compensatory mechanisms might affect the sagittal spinopelvic alignment in various ethnicities. Spinopelvic sagittal alignment and parameters are getting increasing recognition of importance in different ethnicities since patient-specific and ethnicity-specific variation in sagittal spinal contour leads to challenges in characterization and quantification of sagittal spinal deformity in various populations [24], which should be considered when evaluating the sagittal plane and surgical correction strategies [23]. Obviously, it is unreasonable to use the mean values of Caucasian populations in our Chinese populations.

Therefore, the objective of this study is to document the sagittal spinopelvic alignment in a large cohort study in asymptomatic Chinese juveniles and adolescents. Meta-analysis was also performed to explore whether these parameters were different in various ethnicities.

## Materials and methods

### Data collection

A total of 656 asymptomatic young volunteers were included in this retrospective study, who visited the outpatient clinic of our hospital for physical examination from January 2013 to August 2018 and met the inclusion and exclusion criteria. The inclusion criteria were as follows: 1). Age ≤ 18 year; 2). With compete whole spine standing lateral X-ray film; 3). No scoliosis or vertebra growth malformation. The exclusion criteria were as follows: 1). Age > 18 year; 2). Patients were diagnosed as any kind of spine deformity; 3). Patients with neck and back pain, tumours or infections, or those who had hip, knee, and/or ankle abnormalities. This study was approved by the Institutional Review Board of the hospital, and the patients in our study provided written informed consent for the study.

**Fig. 1:**
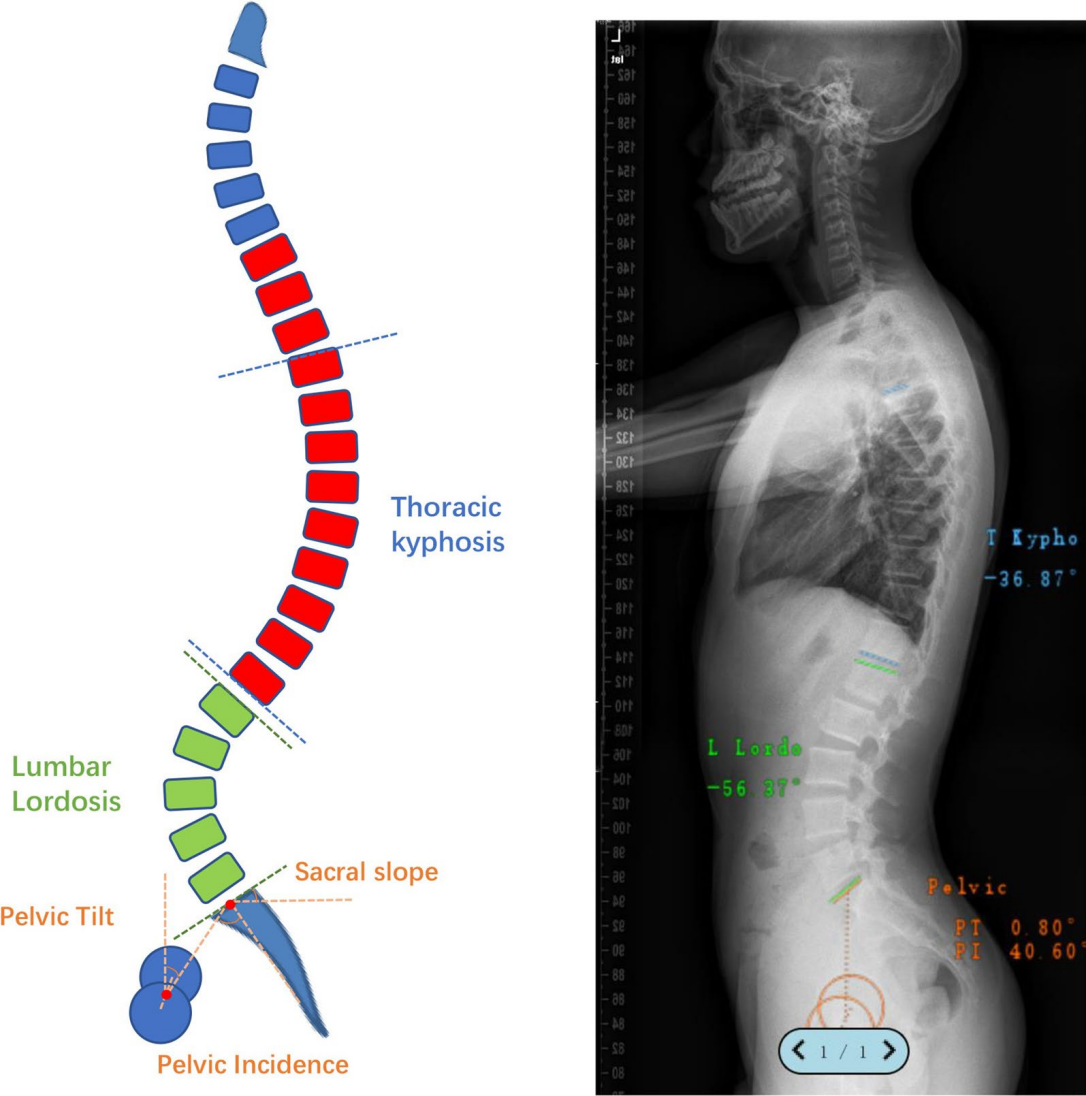
The illustrations of sagittal parameters including TK, LL, PT, PI and SS. As showed in left graph, TK (thoracic kyphosis) was measured as the cobb angle between the upper endplate of T4 vertebra and the lower endplate of T12 vertebra. TLJA (Thoracolumbar junctional angle) was the cobb angle between the upper endplate of T10 vertebra and the lower endplate of L2 vertebra. LL (lumbar lordosis) was the cobb angle between the upper endplate of L1 vertebra and the lower endplate of S1 vertebra. SS (sacrum slope) was the angle between the horizontal and the sacral plate. PT (pelvic tilt) was the angle between the vertical and the line through the midpoint of the sacral plate to femoral heads axis. PI (pelvic incidence) was measured as the angle subtended by a perpendicular from the upper endplate of S1 and a line connecting the center of the femoral head to the center of the upper endplate of S1. The right graph indicated the measurement of TK, LL, PT and PI by using Surgimap software

Demographic data including sex and age were recorded. Lateral radiograph of whole spine standing lateral X-ray film was carried out while maintaining the neck and head in neutral relaxed position to largely eliminate the impacts of postural angles on measurements of spinopelvic parameters. Radiographic parameters were measured by three individual surgeons using Surgimap software, including Risser sign, TK (thoracic kyphosis, cobb angle between the upper endplate of T4 vertebra and the lower endplate of T12 vertebra), LL (lumbar lordosis, cobb angle between the upper endplate of L1 vertebra and the lower endplate of S1 vertebra), TLJA (thoracolumbar junctional angle, cobb angle between the upper endplate of T10 vertebra and the lower endplate of L2 vertebra), SS (sacrum slope, the angle between the horizontal and the sacral plate), PT (pelvic tilt, the angle between the vertical and the line through the midpoint of the sacral plate to femoral heads axis), and PI (pelvic incidence, angle subtended by a perpendicular from the upper endplate of S1 and a line connecting the center of the femoral head to the center of the upper endplate of S1), SVA (sagittal vertical axis, the horizontal offset from the posterosuperior corner of S1 to the vertebral body of C7). PI-LL was calculated by relative PI value minus LL value. The illustrations of sagittal parameters were shown in Fig. 1.

According to the age, asymptomatic young volunteers were divided into two groups: juvenile group (4 < age ≤ 9 years, *n* = 98) and adolescents (10 < age ≤ 18 years, *n* = 558), and sagittal parameters were compared between two groups.

### Meta-analysis

#### Data sources and searches

A systematic online search using PubMed, EMBASE, Web of Science, the Cochrane Library, and China WeiPu Library was performed to identify eligible studies investigating the mean values of sagittal parameters in pre-adulthood populations. The searching strategies were used as follows: (Child OR Children OR Juvenile) OR (Adolescents OR Adolescence) OR (Teens OR Teenager OR Youth) AND (Sagittal). Then, stepwise screening was performed by two authors according to the inclusion and exclusion criteria. There was no limit of language restrictions in the searching progress. Further searches of eligible studies were conducted by searching the reference lists of the selected studies, reviews, or comments.

The inclusion criteria of recruited studies in our meta-analysis were as follows: 1). case–control or cohort studies; 2). concerned with sagittal parameters of child or adolescent; 3). studies with sufficient data. 4). all the studies should report the ethnicities of their study population.

#### Quality assessment and data extraction

Newcastle Ottawa Quality Assessment Scale (NOQAS) [25] was used to assess the quality of all studies. Only studies with a score above 4 were included.

Valuable data from the eligible studies were extracted by two authors, and a consensus was reached by discussion. General characteristics and mean values of sagittal parameters were collected and analyzed.

### Statistical analysis

Statistical analysis was performed using SPSS 19.0 statistics software (SPSS Inc, Chicago, IL). Descriptive statistics were listed in the form of mean ± standard deviation (SD). Radiographic sagittal parameters for juvenile and adolescent group were compared using independent samples t test.

Weight mean difference (WMD) with 95%CI was used to explore the pooled results of sagittal parameters. Sub-group analyses were also performed according to ethnicity rather than the institutions where these study populations received their examinations. Furthermore, volunteers were also divided into two groups according to the age, and meta-analyses were performed in juveniles and adolescents, respectively. The heterogeneity of included studies was examined by a chi-squared-based Q statistical test and quantified by *I*^2^ metric value. If *I*^2^ value was more than 50% or *P* < 0.10, WMD were pooled by the random effect model; otherwise, the fixed effect model was used. Sensitivity analysis was performed to assess the impact of each study on the combined effect of the present meta-analysis. Publication bias was also performed to detect publication bias existed in this study.

Revman 5.3 software was employed and a *P* < 0.05 was considered as statistically significant.

## Results

### Assessment of sagittal spinopelvic alignment

A total of 656 asymptomatic Chinese juveniles (*n* = 98) and adolescents (*n* = 558) were recruited in study. The mean age of the asymptomatic volunteers was 13.14 ± 3.41 years old, including 254 male and 402 female volunteers. The mean values of all the spinopelvic parameters were listed in Table 1. Risser sign in adolescents was significantly greater than that in juveniles (*P* < 0.001); and we also found significant differences of PI (34.20 ± 4.00 vs. 43.18 ± 7.12, *P* < 0.001) and PT (3.99 ± 6.04 vs. 8.42 ± 7.08, *P* < 0.001) between juveniles and adolescents. However, no significant differences of TK, TLJA, LL, SS, PI-LL and SVA were observed between these two groups (all *P* > 0.05, Table 1).

**Table 1:** Mean values of sagittal parameters in different age cohorts

Parameter	All subjects (*n* = 656)	Juveniles (*n* = 98)	Adolescents	*P* value
Age (years)	13.14 ± 3.41	7.03 ± 1.42	14.22 ± 2.37	** < 0.001**
Gender (F/M)	402/254	43/55	359/199	**–**
Risser sign	2.92 ± 1.89	0	3.43 ± 1.57	** < 0.001**
TK (°)	30.41 ± 10.44	32.19 ± 10.77	30.10 ± 10.35	0.798
TLJA (°)	3.71 ± 7.55	2.31 ± 7.79	3.95 ± 7.49	0.806
LL (°)	49.95 ± 9.04	46.34 ± 8.94	50.59 ± 8.92	0.945
PI (°)	41.84 ± 7.47	34.20 ± 4.00	43.18 ± 7.12	** < 0.001**
PT (°)	7.76 ± 7.11	3.99 ± 6.04	8.42 ± 7.08	**0.012**
SS (°)	34.08 ± 6.49	30.21 ± 6.20	34.76 ± 6.31	0.480
PI-LL (°)	− 8.12 ± 9.94	− 12.14 ± 9.76	− 7.41 ± 9.81	0.576
SVA (mm)	− 0.01 ± 21.57	0.83 ± 21.96	− 0.16 ± 21.52	0.761

Bold values indicate a statistical difference

Radiographic sagittal parameters for juvenile and adolescent group were compared using independent samples t test. F meant female, while M meant male

### Meta-analysis

#### Study selection and characteristics

**Fig. 2:**
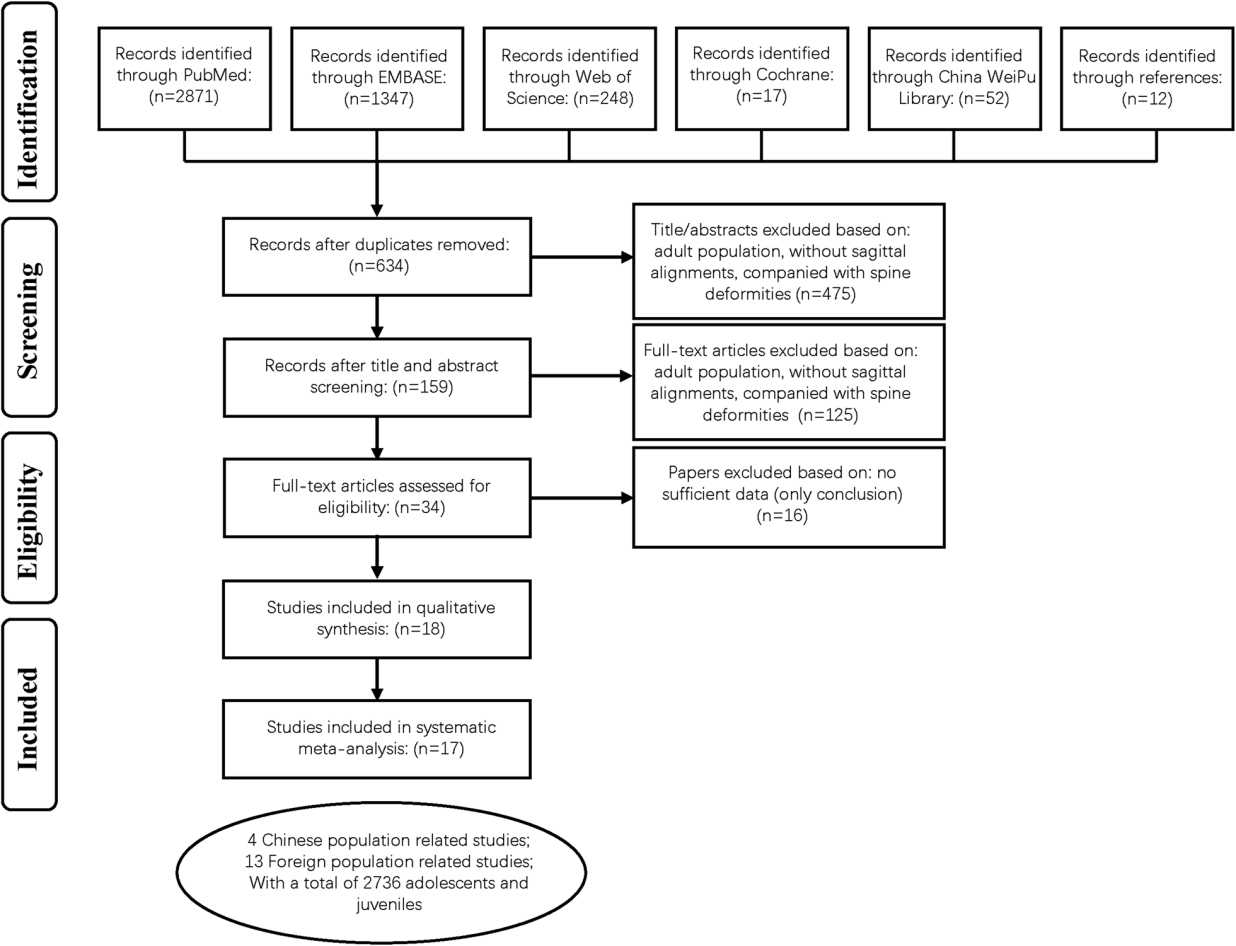
Flow chart showing the process of selection

A total of 17 studies [4–20] were finally recruited in our study, with 6 studies reported in Asian populations [4–9] and 11 studies reported in Caucasians populations [10–20]. The selection process was shown in Fig. 2. The characteristics of these studies were shown in Table 2.

**Table 2:** Characteristics of included studies in our meta-analysis

Author	Year	Country	Ethnicity	N	Age (years)	TK (°)	LL (°)	PI (°)	PT (°)	SS (°)
*Juveniles*										
Our results	2019	China	Asian	98	7.03 ± 1.42	32.19 ± 10.77	46.34 ± 8.94	34.20 ± 4.00	3.99 ± 6.04	30.21 ± 6.20
Descamps et al.	1999	France	Caucasian	29	1–10	38.3 ± 9.8	45.6 ± 12.1	41.8 ± 8.0	NR	40.3 ± 8.7
Mac-Thiong et al.	2004	Canada	Caucasian	35	7.3 ± 1.8	NR	49.2 ± 12.4	44.6 ± 10.6	4.3 ± 8.1	NR
Mac-Thiong et al.	2011	Canada	Caucasian	167	8.1 ± 2.0	42.0 ± 10.6	53.8 ± 12.0	43.7 ± 9.0	5.5 ± 7.6	38.2 ± 7.7
*Adolescents*										
Our results	2019	China	Asian	558	14.22 ± 2.37	30.10 ± 10.35	50.59 ± 8.92	43.18 ± 7.12	8.42 ± 7.08	34.76 ± 6.31
Qiu et al.	2012	China	Asian	33	13.6 ± 2.1	20.8 ± 7.8	49.3 ± 9.9	44.6 ± 11.5	11.3 ± 10.8	33.3 ± 8.2
Zhu et al.	2014	China	Asian	98	14.5 ± 1.5	NR	48.76 ± 10.2	41.2 ± 9.6	6.6 ± 8.1	34.5 ± 9.6
Zhu et al.	2014	China	Asian	90	13.9 ± 2.0	28.7 ± 11.0	46.6 ± 9.8	42.6 ± 9.3	9.9 ± 8.3	32.8 ± 7.1
Liu et al.	2018	China	Asian	60	10–18	24.8 ± 8.8	50.8 ± 10.7	38.3 ± 10.9	3.1 ± 9.4	35.2 ± 8.1
Hiyama et al.	2016	Japan	Asian	24	12–18	21.3 ± 7.6	40.9 ± 11.8	NR	NR	28.5 ± 8.3
Descamps et al.	1999	France	Caucasian	27	10–17	NR	NR	46.8 ± 11.2	NR	NR
Hanson et al.	2002	America	Caucasian	20	11.8	NR	52.1 ± 12.0	47.4 ± 7.5	NR	NR
Mac-Thiong et al.	2004	Canada	Caucasian	145	13.1 ± 2.1	44.2 ± 10.3	49.2 ± 12.4	49.3 ± 11.2	7.9 ± 7.7	41.4 ± 8.5
Upasani et al.	2007	America	Caucasian	50	13.5 ± 2.0	27.9 ± 7.9	55.1 ± 11.9	45.5 ± 8.5	8.4 ± 6.7	37.1 ± 8.5
Mac-Thiong et al.	2011	Canada	Caucasian	479	13.6 ± 1.9	44.8 ± 10.4	57.7 ± 11.1	46.9 ± 11.4	7.7 ± 8.3	39.1 ± 7.6
Schlösser et al.	2013	Holland	Caucasian	95	13.0 ± 1.8	34.9 ± 9.4	53.7 ± 10.1	43.3 ± 12.9	5.6 ± 8.3	37.7 ± 8.6
Ghandhari et al.	2013	Iran	Caucasian	98	13.6 ± 2.9	47.5 ± 12.7	39.6 ± 12.4	45.4 ± 10.7	10.3 ± 6.5	35.4 ± 8.1
Saba Pasha et al.	2014	Canada	Caucasian	35	10–18	44.0 ± 8.0	32.0 ± 15.0	48.0 ± 9.0	12.0 ± 7.0	38.0 ± 12.0
Ries et al.	2015	America	Caucasian	32	15.1 ± 1.9	23.5 ± 8.5	58.2 ± 11.9	48.8 ± 13.1	8.9 ± 9.5	NR
Alzakri et al.	2019	France	Caucasian	51	16.31 ± 1.7	30.21 ± 10.6	52.11 ± 12.0	49.71 ± 11.4	9.61 ± 7.6	40.01 ± 9.4
*Pre-adults*										
Lee et al.	2012	Korea	Asian	181	11.7 ± 4.4	33.2 ± 9.0	NR	NR	9.4 ± 6.1	34.9 ± 6.6
Mac-Thiong et al.	2004	Canada	Caucasian	180	12.0 ± 3.1	43.0 ± 10.4	48.5 ± 12.4	48.4 ± 11.2	7.2 ± 7.9	41.2 ± 8.5
Mac-Thiong et al.	2007	Canada	Caucasian	341	12.1 ± 3.3	44.0 ± 10.9	48.0 ± 11.7	49.1 ± 11.0	7.7 ± 8.0	41.4 ± 8.2
Mac-Thiong et al.	2011	Canada	Caucasian	646	12.1 ± 3.1	44.8 ± 10.6	56.7 ± 11.4	46.0 ± 10.9	5.2 ± 8.2	38.9 ± 7.6

Bold values indicate a statistical difference

### Quality assessment

We used the NOQAS [25] to assess the quality of recruited studies, and the results were shown in Table 3, indicating that the quality of each study was relatively high.

**Table 3:** The quality assessment according to the Newcastle Ottawa Quality Assessment Scale (NOQAS) of each study

Study	Year	Selection	Comparability	Exposure	Total Score
Our study	2021	4	2	3	9
Descamps et al.	1999	3	2	2	7
Hanson et al.	2002	3	2	2	7
Mac-Thiong et al.	2004	3	2	3	8
Mac-Thiong et al.	2007	4	2	3	9
Upasani et al.	2007	4	2	2	8
Mac-Thiong et al.	2011	4	2	3	9
Qiu et al.	2012	3	2	2	7
Lee et al.	2012	4	2	3	9
Schlösser et al.	2013	4	2	2	8
Ghandhari et al.	2013	4	2	3	9
Pasha et al.	2014	3	2	2	7
Zhu et al.	2014	4	2	3	9
Zhu et al.	2014	4	2	3	9
Ries et al.	2015	3	2	2	7
Hiyama et al.	2016	3	2	2	7
Liu et al.	2018	4	2	3	9
Alzakri et al.	2019	4	2	3	9

Newcastle Ottawa Quality Assessment Scale (NOQAS)25 was used to assess the quality of all studies. Only studies with a score above 4 were included. Valuable data from the eligible studies were extracted by two authors, and a consensus was reached by discussion

### Meta-analysis of sagittal parameters in juveniles

3 studies [10, 11, 13] reported TK, LL, PI, PT and SS in juveniles, and these studies were performed in Caucasians. To our knowledge, no study has been performed to explore the normal values of sagittal parameters in asymptomatic Chinese juveniles and our study was the first large-scale research. Therefore, we used our results mentioned above to detect whether there was significant difference of sagittal parameters between Chinese and Caucasian juveniles. Our meta-analysis showed that TK, PI and SS were significant larger in Caucasians populations than those in our study, while we did not find significant differences in LL and PT between these two groups (all *P* > 0.05, Table4).

**Table 4:** Comparisons of sagittal parameters between our results, other Asians and Caucasians

Variables	Test of difference		Model	Test of heterogeneity	
	WMD (95% CI)	*P* value		*P* value	*I*^2^ (%)
Juveniles (our study *vs.* Caucasians)					
TK (°)	**8.18 (4.62, 11.73)**	** < 0.001**	**R**	**0.115**	**59.8**
LL (°)	2.86 (− 2.04, 7.76)	0.525	R	0.006	80.2
PI (°)	**8.22 (6.91, 9.54)**	** < 0.001**	**F**	**0.246**	**28.7**
PT (°)	1.15 (− 0.24, 2.55)	0.105	F	0.522	0
SS (°)	**7.28 (5.86, 8.70)**	** < 0.001**	**F**	**0.264**	**19.8**
Adolescents					
Asians					
TK (°)	− **5.98 (**− **9.47,** − **2.48)**	**0.001**	**R**	** < 0.001**	**85.4**
LL (°)	− **2.94 (**− **5.42,** − **0.45)**	**0.021**	**R**	**0.005**	**73.3**
PI (°)	− 2.18 (− 4.53, 0.17)	0.069	R	0.017	70.4
PT (°)	− 1.19 (− 4.57, 2.19)	0.490	R	< 0.001	88.9
SS (°)	− 1.72 (− 3.51, 0.07)	0.060	R	0.014	68.2
Caucasians					
TK (°)	**7.27 (1.28, 13.27)**	**0.017**	**R**	** < 0.001**	**98.3**
LL (°)	− 0.50 (− 5.21, 4.20)	0.833	R	< 0.001	96.4
PI (°)	**3.89 (2.88, 4.91)**	** < 0.001**	**R**	**0.111**	**37.1**
PT (°)	0.28 (− 0.96, 1.52)	0.656	R	< 0.001	75.6
SS (°)	**3.86 (2.36, 5.35)**	** < 0.001**	**R**	** < 0.001**	**78.8**
Total populations					
TK (°)	2.83 (− 2.99, 8.66)	0.340	R	< 0.001	98.7
LL (°)	− 1.47 (− 4.74, 1.81)	0.380	R	< 0.001	95.2
PI (°)	**2.21 (0.40, 4.02)**	**0.017**	**R**	** < 0.001**	**86.6**
PT (°)	− 0.19 (− 1.44, 1.07)	0.770	R	< 0.001	82.0
SS (°)	1.47 (− 0.44, 3.39)	0.132	R	< 0.001	91.9
Pre-adults					
Asians					
TK (°)	**2.79 (1.26, 4.33)**	** < 0.001**	NR	NR	NR
LL (°)	NR	NR	NR	NR	NR
PI (°)	NR	NR	NR	NR	NR
PT (°)	**1.64 (0.60, 2.68)**	**0.002**	NR	NR	NR
SS (°)	0.82 (− 0.26, 1.90)	0.138	NR	NR	NR
Caucasians					
TK (°)	**13.76 (12.97, 14.55)**	** < 0.001**	**F**	**0.222**	**33.5**
LL (°)	1.14 (− 5.05, 7.34)	0.718	R	< 0.001	98.2
PI (°)	**5.49 (3.85, 7.13)**	** < 0.001**	**R**	** < 0.001**	**83.4**
PT (°)	− 1.09 (− 2.76, 0.58)	0.199	R	< 0.001	87.4
SS (°)	**6.38 (4.60, 8.15)**	** < 0.001**	**R**	** < 0.001**	**89.1**
Total populations					
TK (°)	**10.85 (5.67, 16.03)**	** < 0.001**	**R**	** < 0.001**	**98.1**
LL (°)	1.14 (− 5.053, 7.338)	0.718	R	< 0.001	98.2
PI (°)	**5.49 (3.85, 7.13)**	** < 0.001**	**R**	** < 0.001**	**83.4**
PT (°)	− 0.40 (− 2.27, 1.47)	0.676	R	< 0.001	92.5
SS (°)	**5.01 (2.26, 7.76)**	** < 0.001**	**R**	** < 0.001**	**96.6**

Bold values indicate a statistical difference

Weight mean difference (WMD) with 95%CI was used to explore the pooled results of sagittal parameters. Sub-group analyses were also performed according to ethnicity rather than the institutions where these study populations received their examinations. Furthermore, volunteers were also divided into two groups according to the age, and meta-analyses were performed in juveniles and adolescents, respectively. The heterogeneity of included studies was examined by a chi-squared-based Q statistical test and quantified by *I*^2^ metric value. If *I*^2^ value was more than 50% or *P* < 0.10, WMD were pooled by the random effect model; otherwise, the fixed effect model was used. Sensitivity analysis was performed to assess the impact of each study on the combined effect of the present meta-analysis

### Meta-analysis of sagittal parameters in adolescents

**Fig. 3:**
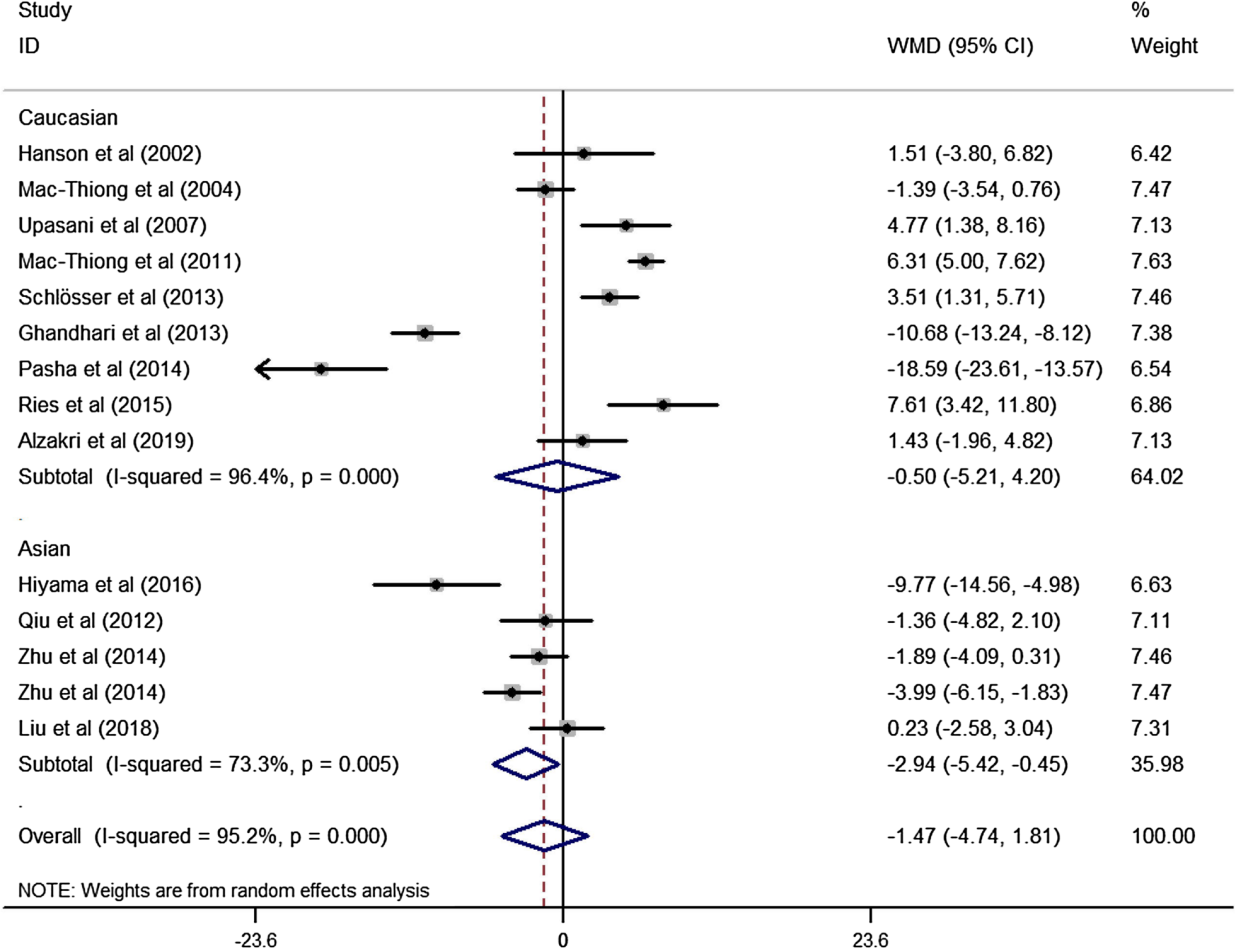
Forest plot describing the meta-analysis for difference of LL of adolescent between our results, other Asian populations and Caucasian populations. LL reported in our study was significantly greater than that reported in other Asian populations; however, we did not find significant difference in LL between our results and Caucasians

**Fig. 4:**
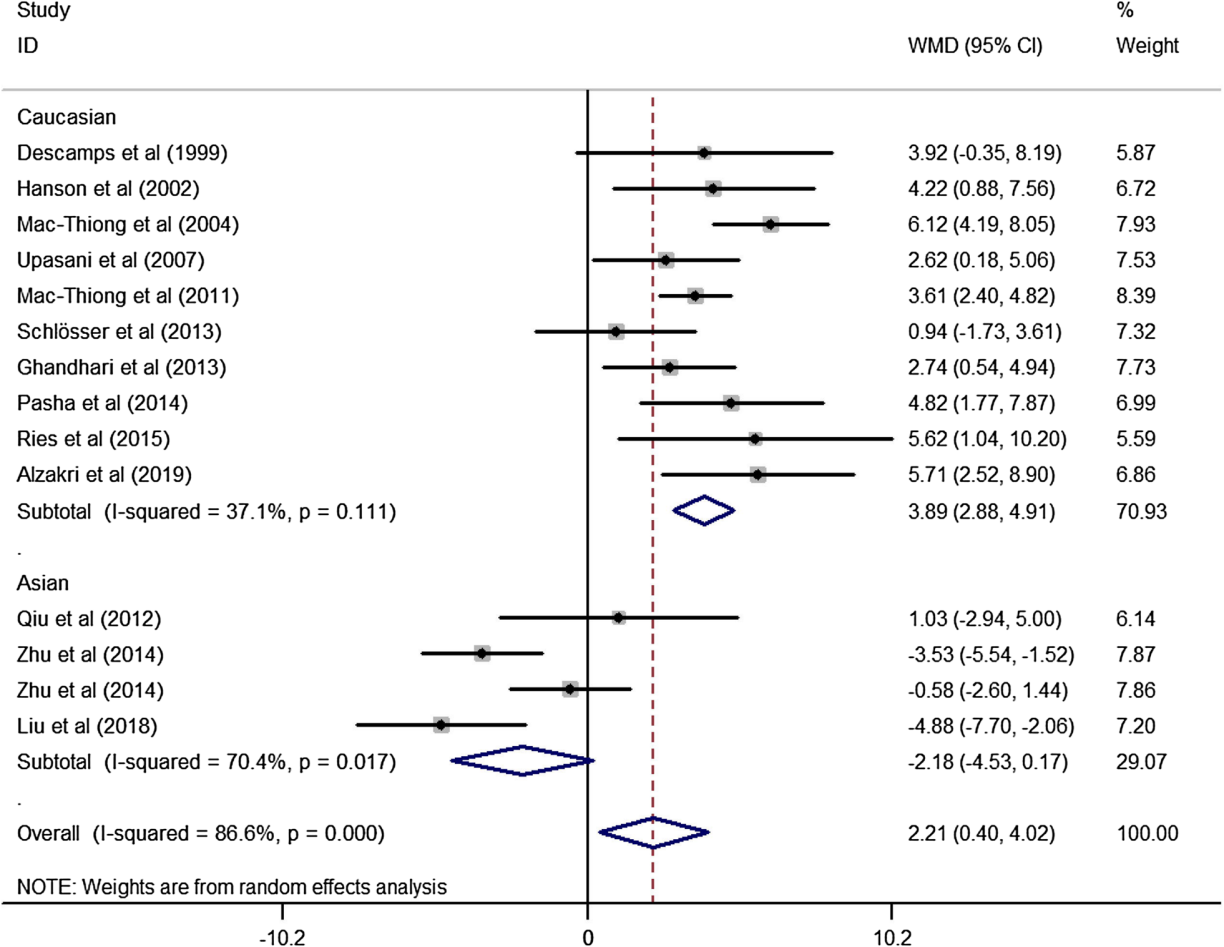
Forest plot describing the meta-analysis for difference of PI of adolescent between our results, other Asian populations and Caucasian populations. PI was found to be greater in Caucasians while no significant difference was observed our results and other Asian populations

A total of 15 studies [4–7, 9–18, 20] reported the sagittal parameters in adolescents, among which 5 studies [4–7, 9] were performed in Asian populations, and other 10 studies [10–18, 20] were performed in Caucasian populations. Combined with our results, the meta-analysis showed that there was significant difference in TK between our populations and other Asian populations; and TK in Caucasians was significantly greater than that in our study. LL reported in our study was significantly greater than that reported in other Asian populations; however, we did not find significant difference in LL between our results and Caucasians. PI was also found to be greater in Caucasians while no significant difference was observed our results and other Asian populations. As to PT, the mean value reported in our study was similar with that reported in other Asian populations and Caucasian populations. SS in Caucasians was significantly greater than that reported in our study, while no significant difference was observed between our study and other Asian populations. All the data were shown in Table 4, Figs. 3 and 4.

### Meta-analysis of sagittal parameters in pre-adults

4 studies [8, 10, 11, 19] reported sagittal parameters in pre-adults whose authors didn’t report the age range of volunteers. Therefore, it was difficult to classify these populations into juvenile or adolescent group. Combined with our results, our study showed that TK and PT in our study were significantly smaller than those in other Asian pre-adults, respectively. However, no significant difference was observed in SS between our cohort group and other Asian pre-adults. No significant data could be used to perform whether there would be difference in LL and PI between our study and other Asian pre-adults. Compared with Caucasian pre-adults, TK, PI and SS in our study were significant smaller (all *P* < 0.001), while no significant difference was observed in LL and PT (all *P* > 0.05). All the data were shown in Table 4.

### Sensitivity analysis and publication bias

We performed a leave-one-out analysis to estimate the sensitivity of our study and found that any single study could be omitted without causing any significant effect on the overall statistical significance, indicating that the results of our meta-analysis were stable. Publication bias was also performed, and we did not find significant publication bias in this study.

## Discussion

To our knowledge, our study was the first large-scale cohort study that reported the mean values of sagittal parameters in asymptomatic Chinese juveniles and adolescents (*n* = 656). In addition, no studies have been performed to document the mean values of sagittal parameters in Asian juveniles, which was crucial to the correction of sagittal alignment in JIS.

Our study was also the first study that reported the norms of sagittal parameters including TK, TLJA, LL, PI, PT, SS, PI-LL and SVA in asymptomatic Chinese juveniles. The mean values of some parameters were inconsistent with previous studies that performed in Caucasian populations [10, 11, 13]. We attributed some reasons to this difference: first, ethnicity might be an important contributor that have a great impact on the skeletal growth and people’s spinopelvic alignment and sagittal parameter [11, 26–28]. In addition to ethnicity, the growth, development and posture of spine and pelvis was also associated with other factors, such as the coordination between spine, pelvis and lower limbs [29], biomechanical factors like walking [30] and life style like prolonged static sitting [31, 32]. Furthermore, our meta-analysis showed that the Caucasian populations tended to have larger PI and SS than Chinese populations, further verifying our previous results. It was important to find out the norm value of sagittal parameters in Chinese populations, rather than using the norm value of other populations when we make surgical planning. Besides, the sample size, measurement errors and the differences of evaluation of sagittal parameters might also contribute to the difference between our study and other studies.

Many studies [4–7, 9–18, 20] have been performed to explore the normal values of sagittal parameters in healthy asymptomatic adolescents with various ethnicities; however, the sample size of these studies was relatively small. Furthermore, although sagittal parameters had been reported in previous studies [10, 11], these normal values could be only used in Canada populations rather than Chinese populations. Although a meta-analysis was performed by Pasha et al. [33]; however, this study aimed to determine the differences in sagittal spinopelvic parameters between adolescent idiopathic scoliosis (AIS) and non-scoliotic controls. In their study, 18 control studies were included, among which, 14 studies were searched out by our searching strategies and the other 4 studies [21, 34–36] were excluded by our inclusion criteria. In addition, sub-group analysis was not performed by ethnicity in their study, which had great influences on sagittal spinopelvic parameters and might cause biases in their results.

A total of 558 asymptomatic Chinese adolescents were recruited in our study. Independent samples *t* test showed that Risser sign, PI and PT were significantly larger in adolescents than those in juveniles, suggesting that skeletal tissues grew and pelvis developed with aging, and pelvis also rotated during the growth to keep the whole sagittal alignment balanced. However, TK, TLJA and LL were not significantly different between juveniles and adolescents, suggesting that spine curves including thoracic kyphosis and lumbar lordosis tended to development maturely in juvenile periods, and less development would occur in adolescent periods. There was no significant difference in PI-LL and SVA between juveniles and adolescents, further indicating the coordinate role of these sagittal parameters in keeping the sagittal balance.

Compared with other Asian adolescent populations [4–7, 9], TK and LL in our study were significantly larger. The selection of study populations, measurement errors and different measurement methods might contribute to these differences. We did not find significant difference in PI, PT and SS between our study and other Asian adolescent populations [4–7, 9], suggesting that pelvis morphology might be similar in Chinese populations and Japanese populations. Furthermore, PI and SS were significant larger in Caucasian population than those in our study, suggesting that Caucasians tended to have larger pelvis morphology and their pelvis might have larger compensation ability to keep sagittal balance. Larger TK was also observed in Caucasian adolescents, indicating that Caucasian populations tended to have larger spine curves and pelvis, which could be classified in Type II sagittal classification proposed by our team [37].

Classification into juvenile and adolescent groups were not performed in some studies [8, 10, 11, 19], and their populations were pre-adults with age < 18 years. We also performed meta-analysis to detect whether these parameters differed differently in pre-adults. The results showed that TK, PI and SS were significantly larger in Caucasians than those in our study, which was consistent with the results in adolescents, further verifying the difference of spine and pelvis morphology between various ethnicities.

Although we have documented the normal values of sagittal alignment in a larger-scale cohort study, some limitations of this study need to be addressed. First, the sample size of juveniles was relatively small compared with adolescent. Second, all the patients recruited in our study were outpatients of a single center. Since multiple minority nationalities such as Bai ethnic minority, Bouyei ethnic minority and Dai ethnic minority exist in China, the conclusion drawn from the study may not be applicable to the Chinese general population. Besides, due to the restriction policy of medical cost, postural angles [22] measured in photogrammetry were not measured and analyzed in this study, which was another limitation. Moreover, on account of little access to BMI, this postural factor was not considered in our study. And because of no information of standardized for the subjects' posture in the included studies, bias may be caused in the comparative results. Therefore, multicenter studies with multiple minority nationalities and measured postural angles should be performed.

## Conclusions

Our study was the first large-scale study that reported the mean values of sagittal parameters in asymptomatic Chinese juveniles and adolescents. Our study indicated that there were significant differences in some parameters between Asian populations and Caucasians, which remind us for using specific mean values in different populations when we restored a relatively normal sagittal spinopelvic balance in spinal deformity.

## Data Availability

The data that support the findings of this study are available from Changhai Hospital, China but restrictions apply to the availability of these data, which were used under license for the current study, and so are not publicly available. Data are however available from the authors upon reasonable request and with permission of Changhai Hospital, China.

## Abbreviations

WMD: Weight mean difference
CI: Confidence interval
HRQoL: Health of Related Quality of Life
TK: Thoracic kyphosis
LL: Lumbar lordosis
PI: Pelvic incidence
PT: Pelvic tilt
SS: Sacrum slope
CSVL: The center sacral vertical line
TLJA: Thoracolumbar junctional angle
SVA: Sagittal vertical axis
PI-LL: Calculation by relative PI value minus LL value
